# Local blood pressure associates with the degree of luminal stenosis in patients with atherosclerotic disease in the middle cerebral artery

**DOI:** 10.1186/s12938-016-0202-1

**Published:** 2016-06-27

**Authors:** Yuanliang Jiang, Wenjia Peng, Zhongzhao Teng, Jonathan H. Gillard, Bo Hong, Qi Liu, Jianping Lu

**Affiliations:** Department of Radiology, Changhai Hospital, Shanghai, 200433 China; Department of Radiology, University of Cambridge, Cambridge Biomedical Campus, Box 218, Cambridge, CB2 0QQ UK; Department of Engineering, University of Cambridge, Cambridge, UK; Department of Neurosurgery, Changhai Hospital, Shanghai, China

**Keywords:** Atherosclerosis, Middle cerebral artery, Blood pressure, Stenosis

## Abstract

The mechanism underlying atherosclerotic ischemic events within the middle cerebral artery (MCA) is unclear. High structural stress induced by blood pressure might be a potential aetiology as plaque rupture occurs when such mechanical loading exceeds its material strength. To perform reliable analyses quantifying the mechanical loading within a plaque, the local blood pressure is needed. However, data on MCA blood pressure is currently lacking. In this study, the arterial pressure proximal to the stenotic site in the MCA was measured in 15 patients scheduled for intervention. The relationships between these local measurements and pre-intervention and intra-intervention non-invasive arm measurements were assessed. The impact of luminal stenosis on the local blood pressure was quantified. Compared with the pre-intervention arm measurement, the intra-intervention arm pressure decreased significantly by 23.9 ± 11.8 and 9.3 ± 14.7 % at diastole and systole, respectively. The pressure proximal to the stenosis was much lower than the pre-intervention arm measurement (diastole: 65.3 ± 15.7 vs 82.0 ± 9.7, p < 0.01; systole: 81.1 ± 15.9 vs 133.9 ± 18.7, p < 0.01; unit: mmHg). The systolic pressure in the MCA in patients with stenosis <70 % (n = 6) was significantly higher than the value in patients with stenosis ≥70 % (n = 9) (92.0 ± 7.3 vs 73.9 ± 16.1, p = 0.02; unit: mmHg), as was pulse pressure (22.8 ± 6.4 vs 11.1 ± 8.3, p = 0.01; unit: mmHg). However, diastolic pressure remained unaffected (69.2 ± 9.3 vs 62.8 ± 19.0, p = 0.58; unit: mmHg). In conclusion, the obtained results are helpful in understanding the local hemodynamic environment modulated by the presence of atherosclerosis. The local pressure measurements can be used for computational analysis to quantify the critical mechanical condition within an MCA lesion.

## Background

Intracranial atherosclerosis has become one of the major subtypes of stroke, accounting for around 8–10 % of strokes in western societies [1] and 33–55 % of strokes in Asian populations [2, 3]. Around 40 % of symptomatic intracranial atherosclerosis is located in the middle cerebral artery (MCA) [4]. The mechanisms underlying stroke related to intracranial atherosclerosis are varied and may include cerebral hypoperfusion, artery-to-artery embolism, plaque extension over small penetrating artery ostia or a combination of aforementioned [5–7]. Difference in stroke pathophysiology may therefore require different preventative and treatment strategies. In current clinical practice, similar to carotid atherosclerotic disease, luminal stenosis is the primary criterion for assessing disease severity in MCA atherosclerosis. Severe luminal stenosis results in flow restriction [8, 9], leading to insufficient cerebral perfusion which has been shown to be predictive for subsequent ischemic events [10]. However, a considerable proportion of culprit MCA lesions (approximately 60 %) are of mild to moderate stenosis (30–69 %) [11, 12]. Further analyses are therefore needed for a better understanding the mechanism underlying the ischemic events caused by MCA atherosclerosis.

Under physiological conditions, atherosclerotic plaques are continually subject to mechanical loading due to blood pressure and flow. Plaque rupture likely occurs when such external loading exceeds its material strength [13, 14]. It has been demonstrated that fibrous cap (FC) rupture is responsible for the majority of ischemic arrests in both carotid [15] and coronary [16] circulations. FC rupture may also be one of the aetiologies for the ischemic events caused by MCA atherosclerosis. In order to perform a reliable estimation for the critical mechanical loading within a MCA atherosclerotic plaque, data are required on lesion geometry, composition, material properties and local blood pressure. However, detailed study of the local blood pressure in MCA with atherosclerosis is currently lacking.

In this study, the local arterial pressure at the location proximal to the MCA stenosis was measured directly. The relationship between this direct measurement and the one obtained from non-invasive arm pressure measurement was assessed and the relationship between the degree of MCA luminal stenosis and the local arterial pressure was explored.

## Methods

Eighteen patients with symptomatic MCA atherosclerotic disease, scheduled for cerebral vascular intervention, were recruited in Changhai Hospital, Shanghai, China. The study was approved by the local ethics committee (Ref: CHEC2013204), with all patients giving written informed consent prior to their procedure. The patient demographics are listed in Table 1.

**Table 1:** Patient demographics (n = 15)

n (%)/mean ± SD	Luminal stenosis		p value
	<70 % (n = 6)	≥70 % (n = 9)	
Sex (male)	2 (33.3)	4 (44.4)	0.14^†^
Age	59.9 ± 9.7	60.5 ± 10.8	0.05^‡^
Arm diastolic pressure (sober; mmHg)	82.1 ± 9.7	82.5 ± 10.9	0.06^‡^
Arm Systolic pressure (sober; mmHg)	133.9 ± 19.1	131.5 ± 19.8	0.58^‡^
Heart rate (sober;/minute)	77.3 ± 4.3	77.5 ± 4.3	0.78^‡^
Hypertension	6 (100)	5 (55.6)	0.60^†^
Atrial fibrillation	0 (0)	0 (0)	N/A
Ischemic heart disease	0 (0)	0 (0)	N/A
Diabetes	3 (50)	4 (44.4)	0.32^†^
High cholesterol	3 (50)	3 (33.3)	0.62^†^
Peripheral vascular disease	0 (0)	0 (0)	N/A
Previous TIA/stroke	6 (100)	4 (44.4)	1^†^
Aspirin used before recruitment	2 (33.3)	3 (33.3)	0.33^†^
Luminal stenosis (mean ± SD)	57.3 ± 10.0 %	78.6 ± 5.1 %	<0.01*

^†^ Fisher’s exact test

^‡^ Student t test

* Mann–Whitney test

**Fig. 1:**
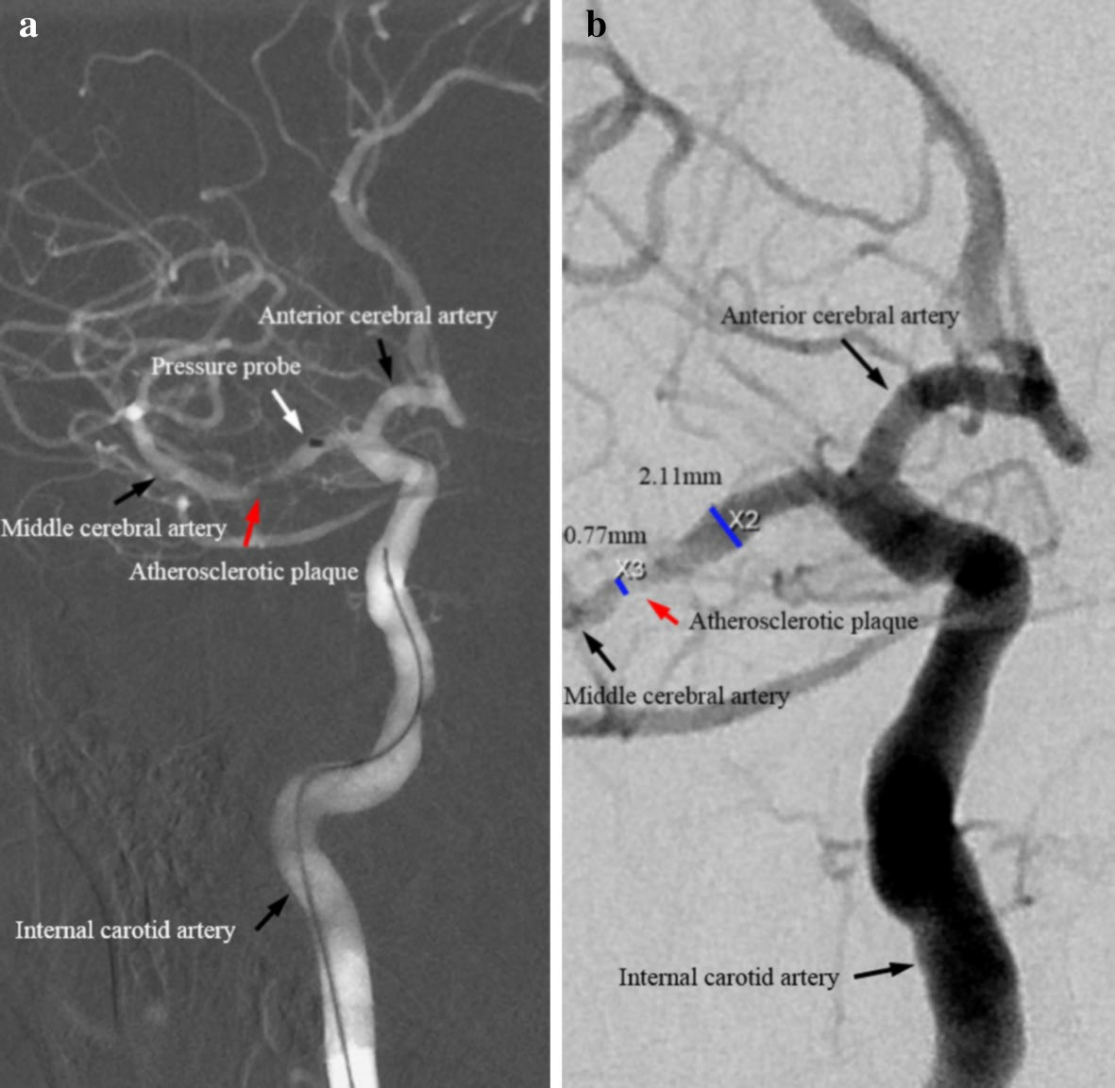
Digital subtraction angiographic images showing an atherosclerotic plaque in the middle cerebral artery (**a** image highlighting a microcatheter (marked by *white arrow*) and an atherosclerotic plaque in the middle cerebral artery (marked by *red arrow*); **b** image illustrating dimensions at the most stenotic site and the proximal disease-free site; **a** and **b** were from the same patient.)

All procedures were performed via femoral access using a 6Fr sheath (Medtronic, Minneapolis, USA), with a bolus dose of intra-arterial unfractionated heparin (80 IU/kg) under a general anesthetic. Ebrantil (Nycomed Deutschland GmbH, Konstanz Germany) was administered to control blood pressure. Selective angiography was performed via a 5Fr pigtail catheter (Medtronic, Minneapolis, USA). A microcatheter (DePuy Orthopaedics, Inc., Warsaw, USA) was placed in the target MCA proximally to the stenotic segment to record the local arterial pressure (Fig. 1) over a 10–15 s period. Attempts were also made to measure the local pressure distally to the stenosis (the measurement at the distal site was only successful in three patients). Both systolic and diastolic values were averaged to calculate the representative values for each patient. Non-invasive upper arm blood pressure was also monitored during the interventional procedure. Luminal stenosis was calculated following WASID method [17] using the ratio between dimensions at the most stenotic site and the reference at the proximal normal section on digital subtraction angiography (DSA) images (Fig. 1).

Categorical variables were analysed using two-sided Fisher’s exact test. Normal distribution was tested by Shapiro–Wilk test. All pressure measurements were not followed the normal distribution. Two-tailed Mann–Whitney test were therefore used. The difference between pressures (pre-intervention vs intra-intervention arm pressure vs MCA pressure) acquired in the same patient was assessed using paired Wilcoxon signed-rank test. Statistical analyses were performed in R 2.10.1 (The R Foundation for Statistical Computing). The regression quantifying the relationship between local pressure and luminal stenosis was performed in Matlab R2014a (MathWorks Inc., USA).

## Results

**Fig. 2:**
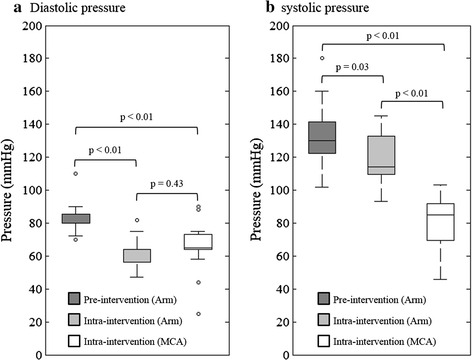
Comparisons between local blood pressure measurement at the site proximal to the atherosclerosis in the MCA and the arm blood pressure measured before the intervention and during the intervention (**a** comparison of pressures at diastole; and **b** comparison of pressure at systole)

The local MCA arterial pressure at the proximal site was recorded successfully in 15 (out of 18) patients. During the procedure, compared with pre-intervention measures, non-invasive arm pressures at diastole and systole decreased by 23.9 ± 11.8 and 9.3 ± 14.7 %, respectively (Fig. 2). The diastolic pressure at the location proximal to the stenosis was comparable with the one obtained at the arm during the intervention (65.3 ± 15.7 vs 62.0 ± 9.4 mmHg, p = 0.43), while the systolic pressure was lower by 32.0 ± 12.0 % compared with the same reference (81.1 ± 15.9 vs 119.7 ± 15.5 mmHg, p < 0.01). The concrete pressure measurement of each patient was listed in Table 2. The local pressure distally to the stenosis was acquired successfully only on P01, P02 and P03 (Table 2) (diastole/systole; P01: 51/42; P02: 35/33; and P03: 25/21; unit: mmHg).

**Table 2:** Blood pressure measurements of each patient

Patient ID	Pre-intervention arm pressure (mmHg)		Intra-intervention arm pressure (mmHg)		Intra-intervention MCA pressure (mmHg)		Stenosis (%)
	Diastole	Systole	Diastole	Systole	Diastole	Systole	
P01	80	160	56	137	67	91	48
P02	110	180	75	131	64	92	62
P03	70	102	64	106	65	92	42
P04	70	120	57	132	65	80	61
P05	80	130	64	145	88	103	64
P06	80	130	64	133	66	94	67
P07	72	144	48	93	64	67	79
P08	80	130	64	135	44	57	78
P09	86	118	64	109	75	85	75
P10	90	150	47	99	25	46	75
P11	80	130	54	113	71	85	76
P12	84	134	71	127	90	93	78
P13	88	130	63	111	64	90	83
P14	80	120	57	114	74	77	74
P15	80	130	82	111	58	65	90

**Fig. 3:**
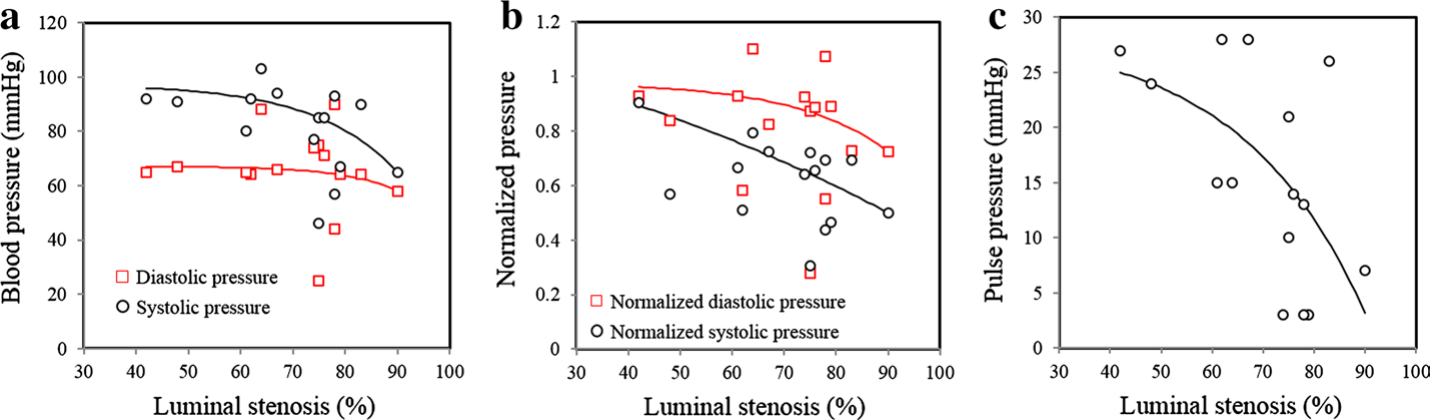
The relationship between local blood pressure in the MCA and luminal stenosis and the corresponding fitting *curve* (**a** local blood pressure vs stenosis; **b** normalized blood pressure vs stenosis (diastolic and systolic pressure in the MCA were normalized by the arm diastolic and systolic pressure measured before intervention); and **c** pulse blood pressure vs stenosis)

As presented in Fig. 3, the degree of luminal stenosis affected both local systolic and pulse pressure (the difference between systolic pressure and diastolic pressure) significantly (Fig. 3a, c). Both of these decreased as MCA stenosis severity increased. The MCA stenosis had very little effect on local diastolic pressure (Fig. 3a). The same trend was observed when the pressure measurements in MCA was normalized by pre-intervention arm pressure (Fig. 3b). The relationship between pressure and the luminal stenosis can be expressed by

$$Pressure = k_{1} e^{{k_{2} \times Stenosis}} + k_{3}$$

in which *k*_1_, *k*_2_ and *k*_3_ are constants. The fitted constants are listed in Table 3. In this study, 9 lesions caused sever (≥70 %) luminal stenosis and 6 caused mild or moderate (30–69 %) luminal stenosis. The systolic and diastolic pressures measured in patients with moderate stenosis were 92.0 ± 7.3 and 69.2 ± 9.3 mmHg, respectively; and in patients with severe stenosis were 73.9 ± 16.1 and 62.8 ± 19.0 mmHg, respectively.

**Table 3:** The fitting constants and the corresponding correlation coefficient describing the relationship between pressure and luminal stenosis

	*k*_1_ (mmHg)	*k*_2_	*k*_3_ (mmHg)	*R* ^2^
Diastolic pressure (mmHg)	−0.0013	0.0982	67.0835	0.02
Systolic pressure (mmHg)	−0.1037	0.0638	97.4290	0.34
Normalized diastolic pressure	−0.0016	0.0562	0.9781	0.19
Normalized systolic pressure	−0.4922	0.0091	1.6144	0.64
Pulse pressure (mmHg)	−0.6541	0.0407	28.6071	0.37

## Discussion

To authors’ best knowledge, this is the first study reporting direct measures of the local blood pressure for MCA atherosclerosis. The obtained results indicate that local arterial pressure is related to the degree of luminal stenosis in the MCA. Notably, the systolic and pulse pressures decreased as the stenosis increased. Considering Laplace’s law as the first-order approximation,

$$\bar{\sigma } = \frac{pr}{h}$$

the mean structural stress ($\bar{\sigma }$) is correlated with local pressure (*p*) and inner radius (*r*) and inversely correlated with wall thickness (*h*). Compared with lesions with severe luminal stenosis, those in the mild to moderate category have a bigger *r* and smaller *h*; as shown in this study, local pressure (*p*) is higher in this category; the structural stress is therefore higher when the luminal stenosis is reduced. Moreover, pressure variation is bigger in the lesion with mild or moderate luminal stenosis, which leads to an increased stress variation. These changes both have significant potential to destabilize the plaque structure [18]. It might therefore be reasonable to suggest that FC rupture more likely occurs in lesions with thin FC but only caused mild to moderate luminal narrowing.

Certainly, mechanical loading in the lesion is not only dominated by local blood pressure, but also morphological and compositional plaque features [19, 20], e.g., it has been shown that mechanical stress within FC increases with local luminal curvature increase [21] and FC thickness decrease [22, 23]. For an accurate stress calculation, sophisticated mechanical analyses are needed that consider 3D plaque geometry, atherosclerotic composition, tissue material properties, and boundary/loading conditions. Pilot results obtained from carotid have demonstrated the complementary values of mechanical analyses in predicting subsequent ischemic events to plaque architectures [24, 25]. Morden advanced magnetic resonance imaging techniques are enable to delineate plaque geometry and compositions in the MCA non-invasively [7, 12]. However, it is challenging to directly measure the local blood pressure in symptomatic patients scheduled for medical therapy or in asymptomatic patients. This study provides useful information to estimate the local MCA blood pressure based on the non-invasive arm measurements and luminal stenosis.

Despite of the great value of the data reported, limitations exist: (1) the patient cohort is small (the local pressure was acquired successfully in 15 patients); and (2) local blood pressure acquired might have been affected by the probe.

## Conclusions

This study indicates that local arterial pressure is related to the degree of luminal stenosis in the MCA. Notably, the systolic and pulse pressures decreased as the stenosis increased. The obtained results are helpful in understanding the local hemodynamic environment modulated by the presence of atherosclerosis and can be used for computational analysis to quantify the critical mechanical condition within an MCA lesion.

## Abbreviations

DSA: digital subtraction angiography
FC: fibrous cap
MCA: middle cerebral artery
